# Increased Serum Leptin Level Predicts Bone Mineral Density in Hemodialysis Patients

**DOI:** 10.1155/2020/8451751

**Published:** 2020-06-03

**Authors:** Chih-Hsien Wang, Yu-Hsien Lai, Yu-Li Lin, Chiu-Huang Kuo, Ru-Jiang Syu, Ming-Chun Chen, Bang-Gee Hsu

**Affiliations:** ^1^Division of Nephrology, Hualien Tzu Chi Hospital, Buddhist Tzu Chi Medical Foundation, Hualien, Taiwan; ^2^School of Medicine, Tzu Chi University, Hualien, Taiwan; ^3^Division of Nephrology, Department of Internal Medicine, Dalin Tzu Chi Hospital, Buddhist Tzu Chi Medical Foundation, Chiayi 62247, Taiwan; ^4^Department of Pediatric, Hualien Tzu Chi Hospital, Buddhist Tzu Chi Medical Foundation, Hualien, Taiwan

## Abstract

**Background:**

Leptin acts through the adipose-bone axis to regulate bone mineral density (BMD). This study evaluated the relationship between BMD and serum leptin levels in patients on hemodialysis.

**Methods:**

In this cross-sectional study including 98 hemodialysis patients, BMD was measured using dual energy X-ray absorptiometry of the lumbar vertebrae (L2–L4), and serum leptin levels were determined using an enzyme immunoassay.

**Results:**

There were 25 (25.5%), 13 (13.3%), and 60 (61.2%) patients with osteopenia, osteoporosis, and normal BMD, respectively. Advanced age (*P*=0.017); decreased body mass index (BMI, *P* < 0.001); body height (*P* < 0.001); prehemodialysis body weight (BW, *P* < 0.001); post-hemodialysis BW (*P* < 0.001); waist circumference (*P* < 0.001); and triglyceride (*P*=0.015), albumin (*P*=0.004), and leptin levels (*P*=0.017) were associated with lower lumbar *T* scores, whereas increased urea reduction rate (URR, *P*=0.004) and fractional clearance index for urea (Kt/V, *P*=0.004) were associated with lower lumbar *T* scores. The multivariable forward stepwise linear regression analysis with adjustment for sex; age; body height; prehemodialysis BW; BMI; waist circumference; logarithmically transformed triglycerides (log-triglycerides), albumin, creatinine, and leptin (log-leptin) levels; URR; and Kt/V indicated that high serum level of log-leptin (*R*^2^ change = 0.184; *P* < 0.001), increased prehemodialysis BW (*R*^2^ change = 0.325; *P*=0.008), male sex (*R*^2^ change = 0.048; *P*=0.001), young age (*R*^2^ change = 0.044; *P*=0.012), and increased serum albumin level (*R*^2^ change = 0.017; *P*=0.044) were significantly and independently associated with lumbar BMD.

**Conclusions:**

Advanced age and female sex were associated with poor BMD, whereas increased BW, serum albumin, and leptin levels were positively associated with BMD in patients on hemodialysis.

## 1. Introduction

Osteoporosis, characterized by low bone mass and density resulting from the imbalance between bone formation and resorption, is a global public health issue associated with an increased risk of fractures [1]. Epidemiological studies have estimated >34 million and 10 million individuals with osteopenia and osteoporosis, respectively, worldwide [2]. Specifically, chronic kidney disease-(CKD-) associated mineral and bone disorder is a complex, multifactorial clinical entity in patients with end-stage renal disease, and bone mineral metabolism gradually deteriorates starting from the early stages of CKD [3]. Among patients on hemodialysis, the reported prevalence rates of osteoporosis in the femoral neck and lumbar spine are approximately 16%–19% and 13%–29%, respectively [4, 5].

Patients with low bone mineral density (BMD) are proposed to be at high risk for osteoporosis and fractures. The factors that can affect BMD are sex, age, physical activity, nutrition, activity of the renin-angiotensin system, and adipokine levels [6, 7]. Leptin, a 16 kDa obesity-associated adipokine with 167 amino acids that is primarily secreted by white adipocytes, was identified in the early 1990s and was shown to play a crucial role in regulating the neuroendocrine axes as well as the glucose and fat metabolism [8]. There is a close relationship between bone and adipose tissue. Previous *in vitro* studies have demonstrated that leptin had an impact on the differentiation and proliferation of osteoblasts and osteoclasts [9–12]. Several studies in humans have showed a positive association between leptin and bone parameters [13, 14]. Also, one recent study revealed that leptin played essential roles in several bone disorders including osteoporosis, fractures, osteoarthritis, and bone tumors [15].

Leptin is typically elevated in patients with renal failure, whereas deterioration of the renal function leads to homoeostatic disturbances in bone and adipose tissue [16, 17]. Although leptin has important roles in bone remodeling, its effect on bone density of patients on hemodialysis is unclear. Therefore, we aimed to evaluate the relationship between BMD and serum leptin levels in patients on hemodialysis in a cross-sectional study.

## 2. Materials and Methods

### 2.1. Patients

Ninety-eight patients on hemodialysis, including 48 males and 50 females (age range, 50–87 years) who received the standard 4-hour dialysis three times per week for at least three months at the hemodialysis unit of Hualien Buddhist Tzu Chi General Hospital between June 2015 and August 2015 were enrolled. All patients were under hemodialysis using the same high-flux polysulfone disposable artificial kidney system (FX class dialyzer, Fresenius Medical Care, Bad Homburg, Germany) with the standard bicarbonate dialysate. This study was approved by the Protection of Human Subjects Institutional Review Board of Hualien Tzu Chi Hospital. The inclusion criteria were as follows: age ≥50 years; those on maintenance hemodialysis for longer than 6 months. The exclusion criteria were as follows: acute myocardial infarction, pulmonary edema, heart failure, acute infection, malignancy at the time of blood sampling, use of osteoporosis drugs (bisphosphonates, teriparatide, or estrogen medications), history of lumbar fracture or surgery, and refusal to provide informed consent for the study. Blood pressure (BP) was measured before hemodialysis by using standard mercury sphygmomanometers with appropriate cuff sizes after the participant was sitting for at least 30 min. Systolic BP (SBP) and diastolic BP (DBP) were taken three times at 5 min intervals and were averaged for analysis. Fractional clearance index for urea (Kt/V) and urea reduction ratio (URR) were measured before and immediately after dialysis using a formal, single-compartment model of dialysis urea kinetics.

### 2.2. Anthropometric Analysis

Body weight (BW) and height were measured with the patient in light clothing and without shoes to the nearest half a kilogram and half a centimeter, respectively. Waist circumference was measured to the nearest half a centimeter at the shortest point below the lower rib margin and the iliac crest. Body mass index (BMI) was calculated using Quetelet's formula as weight (kg) divided by height squared (m^2^) [8, 18, 19].

### 2.3. Biochemical Analyses

Approximately 5 ml of the blood sample was collected before hemodialysis from all participants. The samples were immediately centrifuged at 3000*g* for 10 min and stored at 4°C within one hour of collection for downstream biochemical analyses. Serum levels of total cholesterol (TCH), triglycerides, albumin, globulin, blood urea nitrogen, creatinine, glucose, total calcium, and phosphorus were measured using an autoanalyzer (Siemens Advia 1800, Siemens Healthcare, Henkestr, Germany). Serum leptin concentrations were measured using a commercially available enzyme immunoassay kit (SPI-BIO, Montigny le Bretonneux, France) [19, 20]. Serum intact parathyroid hormone (iPTH) levels were measured using an autoanalyzer (Siemens Advia Centaur XP immunoassay system, Siemens Healthcare).

### 2.4. Bone Mineral Density Measurements

After blood sampling, the patients were evaluated for BMD measurements. BMD for the lumbar vertebrate (L2–L4) was measured using dual energy X-ray absorptiometry (QDR 4500, Hologic, MA, USA). BMD measurements were expressed as absolute values (g/cm^2^), *Z* scores, and *T* scores (deviation from peak BMD) [18]. *Z* score was defined as the number of standard deviations from the mean BMD of age-, weight-, and ethnicity-matched healthy populations. *T* score was defined as the number of standard deviations from the mean BMD for sex-matched young normal controls. Compared to the control value, a lumbar bone *T* score lower than −2.5 was used as the diagnostic cutoff for osteoporosis, and a lumbar bone *T* score of −1.0 to −2.5 was used for the diagnosis of osteopenia, according to World Health Organization criteria.

### 2.5. Statistical Analysis

All statistical analyses were performed using Statistical Package for the Social Sciences (SPSS) (version 19.0; SPSS, Chicago, IL, USA). Data were tested for normal distribution using the Kolmogorov–Smirnov test. Normally distributed data were presented as means ± standard deviation and tested with the two-tailed independent *t*-test, and nonnormal distributed data were expressed as medians with interquartile ranges. Significance of differences among groups (normal, osteopenia, and osteoporosis) was determined using the Kruskal–Wallis test for parameters that had non-normal distributions and by the one-way analysis of variance for normally distributed data. Data based on the number of patients were expressed as the percentage (%) of study population and evaluated by the chi-square test. The data on triglycerides, glucose, iPTH, and leptin showed skewed nonnormal distributions and were therefore recalculated by logarithmic transformation to the base 10; after the transformation, log-triglycerides, log-glucose, log-iPTH, and log-leptin showed normal distribution. Clinical variables that correlated with lumbar BMD in patients on hemodialysis were evaluated by the simple linear regression analysis followed by the multivariable forward stepwise regression analysis. The receiver operating characteristic (ROC) curve was used to calculate the area under the curve (AUC) to identify the most proper cutoff value of leptin to predict lumbar *T* score cutoff values in hemodialysis patients. A *P* value <0.05 was considered statistically significant for all analyses.

## 3. Results

The clinical and laboratory characteristics of the hemodialysis patients included in the current study are presented in Table 1. Briefly, 25 (25.5%) and 13 (13.3%) patients on hemodialysis had osteopenia and osteoporosis, respectively. Among the group with lower bone density, decreased serum leptin (*P*=0.017), triglyceride (*P*=0.015), and albumin (*P*=0.044) levels, older age (*P*=0.017), lower body height (*P* < 0.001), pre- and postdialysis BW (both *P* < 0.001), waist circumference (*P* < 0.001), BMI (*P* < 0.001), lumbar BMD (*P* < 0.001), lumbar *T* and *Z* scores (both *P* < 0.001), increased URR (*P*=0.004), Kt/V (*P*=0.004), and female hemodialysis patients (*P* < 0.001) were significantly correlated with lower lumbar *T* score cutoff values. The ROC curve analysis showed that the best cutoff serum value of leptin to predict lumbar *T* score cutoff values in hemodialysis patients was 5.92 ng/mL with AUC 0.665 (95% confidence interval, 0.554–0.763; *P*=0.010), sensitivity 44%, and specificity 85%, respectively (data not shown).

Sex, comorbidities, drugs use, and lumbar BMD values of the patients on hemodialysis patients are presented in Table 2. Briefly, the cohort comorbidities included diabetes (*n* = 47; 48.0%) and hypertension (*n* = 49; 50.0%). There were 21 (21.4%), 32 (32.7%), 34 (34.7%), 16 (16.3%), and 9 (9.2%) patients using angiotensin-converting enzyme inhibitors or angiotensin receptor blockers, *β*-blockers, calcium channel blockers, statins, and fibrates, respectively. Female hemodialysis patients had lower lumbar BMD than male hemodialysis patients (*P* < 0.001). The mean rates for comorbid conditions of diabetes and hypertension and the mean rates for the use of angiotensin-converting enzyme inhibitors or angiotensin receptor blockers, *β*-blockers, calcium channel blockers, statins, and fibrates were similar between the two groups.

The simple linear analysis and multivariable forward stepwise linear regression analysis of the lumbar BMD in 98 hemodialysis patients is presented in Table 3. Height (*r* = 0.531, *P* < 0.001), predialysis BW (*r* = 0.570, *P* < 0.001), WC (*r* = 0.429, *P* < 0.001), BMI (*r* = 0.418, *P* < 0.001), log-triglycerides (*r* = 0.220, *P*=0.029), albumin (*r* = 0.239, *P*=0.018), creatinine (*r* = 0.220, *P*=0.029), and log-leptin (*r* = 0.568, *P* < 0.001) were positively correlated with lumbar BMD in patients on hemodialysis. Conversely, female sex (*r* = −0.490, *P* < 0.001), age (*r* = −0.321, *P*=0.001), URR (*r* = −0.382, *P* < 0.001), and Kt/V (*r* = −0.380, *P* < 0.001) were negatively correlated with lumbar BMD in patients on hemodialysis. After the multivariable forward stepwise linear regression analysis of the variables that were significantly associated with lumbar BMD (female sex, age, height, prehemodialysis BW, waist circumference, BMI, log-triglycerides, albumin, creatinine, log-leptin, URR, and Kt/V), log-leptin (*R*^2^ change = 0.184; *P* < 0.001), prehemodialysis BW (*R*^2^ change = 0.325; *P*=0.008), albumin (*R*^2^ change = 0.017; *P*=0.044), female sex (*R*^2^ change = 0.048; *P*=0.001), and age (*R*^2^ change = 0.044; *P*=0.012) were independent predictors of lumbar BMD among the hemodialysis patients.

## 4. Discussion

The current cross-sectional study revealed that lumbar BMD had a significant positive association with serum leptin and albumin levels and BW and that advanced age and female sex were associated with poor BMD among the hemodialysis patients.

There is a high prevalence of BMD reduction on maintenance dialysis patients, with more prominent in patients on hemodialysis than those on peritoneal dialysis [3, 5]. The etiology of osteoporosis in hemodialysis patients is multifactorial, and several risk factors in patients with end-stage renal disease were reported [6, 21, 22]. Our data revealing significant positive associations of increased BW and serum albumin level and significant negative associations of advanced age and female sex with BMD agree with these previous studies.

Many studies have attempted to evaluate the correlation of BMD with clinical or laboratory factors on maintenance dialysis patients; however, the results were controversial because of the conflicting findings [22]. Serological markers of bone turnover utilized to predict the nature of renal osteodystrophy, low bone mass, and fracture rates have not been well established in hemodialysis populations [23]. The present study also showed no significant differences in serum calcium, phosphorus, or iPTH levels among hemodialysis patients with osteopathy or those with normal BMD values. These laboratory parameters to determine bone turnover in serum samples, which can be influenced by many therapeutic interventions, are therefore not ideal markers, highlighting the need for new markers to assess osteoporosis in hemodialysis patients [22].

Beyond the traditional risk factors, accumulating evidences reveal the cross-talk between adipocytes and bony tissues and that adipokines exert direct or indirect effects on bone metabolism [13, 14]. Prior cross-sectional reports represented that leptin was positively associated with BMD [13, 14, 24]. Specifically, BMD was increased in parallel with increasing leptin concentrations in premenopausal and postmenopausal females as well as in males in a cohort of 5,815 American adults [25]. Yamauchi et al. reported that circulating leptin levels exhibited a significant positive correlation with BMD at all the skeleton sites measured in 139 postmenopausal females and that this positive association was conserved with whole-body BMD values even after age and fat ratio were taken into consideration in the multiple regression analysis [24]. A recent systemic review and meta-analysis suggested that circulating leptin levels were positively associated with BMD and bone mineral content, especially in postmenopausal females. Besides, plasma leptin levels were positively correlated with lumbar spine BMD in both premenopausal and postmenopausal females [26]. Ghazali et al. demonstrated that leptin levels correlated with BMD in the femoral neck of male hemodialysis patients [27]. These data altogether provide convincing evidence that serum leptin levels are significantly correlated with BMD in different populations, and that leptin might be involved in the progression of osteoporosis.

Serum leptin increases bone mass via interaction with bone marrow mesenchymal stem cells (BMSCs), osteoclasts, osteoblasts, and chondrocytes [28]. *In vitro*, leptin stimulates the proliferation and differentiation of BMSCs to osteoblasts rather than adipocytes [10, 29]. In addition, leptin also inhibits osteoclastogenesis by increasing osteoprotegerin expression and decreasing the synthesis of receptor activator of nuclear factor kappa-Β (RANK) ligand in stromal cells [11]. *In vivo*, leptin increases the proliferation of fetal rat osteoblasts in bone and inhibits osteoclast generation in bone marrow, leading to the new bone formation with higher bone density and reduced fracture risk [9]. Moreover, systemic leptin treatment reverses poor fracture healing in leptin-deficient ob/ob mice [30] and increases bone growth following ovariectomy-induced bone loss in mice [31]. Leptin administration also promotes chondrocyte proliferation and increases the insulin-like growth factor-1 receptor expression in the cartilage of the growth plate and mandibular condyle [15]. Through the activation of fibroblast growth factor-23, leptin may also influence bone growth [32]. A recent study also stated that osteoblasts and growth plate cartilage are positively regulated by leptin through peripheral signaling rather than the hormone's CNS-mediated anorexigenic actions [33]. Overall, peripheral leptin is considered to exert a direct anabolic activity on bone metabolism in contrast with central antiosteogenic effects [34, 35].

Besides, leptin also acts indirectly to promote bone growth. Studies revealed that serotonin could bind to the serotonin 1b receptor on osteoblasts to inhibit bone growth [36, 37], whereas leptin was shown to inhibit serotonin synthesis and decrease serotonergic receptors, leading to improved bone growth [36]. Similar to estrogen, leptin also increases osteoprotegerin levels and activate the RANK ligand-binding receptor, which may result in reduced osteoclast activity [38]. Furthermore, leptin was demonstrated to inhibit high glucocorticoids, and cortisol levels induced decreased bone mass and osteocalcin through the hypothalamic-pituitary-adrenal axis and, in turn, stimulate bone growth [39]. In conclusion, leptin has important direct and indirect effects on bone growth and metabolism via distinct pathways.

Kidneys play a significant role in removing circulating leptin, and serum leptin levels increase with the CKD progression even after correction for age and body fat mass [16, 40]. Serum leptin is not cleared by hemodialysis, and Polymeris et al. reported increased serum leptin levels in hemodialysis patients in accordance with the findings of the present study [41, 42], illustrating the potential role of hyperleptinemia in BMD among patients on the maintenance dialysis.

There are several limitations in the current study. First, this was a cross-sectional study without a control group and body composition, inflammatory, and nutritional status, which have the influence on serum leptin level among HD patients not accessed, may pose some bias in our study [20]. Furthermore, prospective studies are necessary to confirm a causal relationship between circulating leptin and BMD in hemodialysis patients. Second, due to the small sample size of the study cohort, a type II error may obscure differences between the normal BMD and osteopenia/osteoporosis groups. Third, the impact of some lifestyle and environmental factors such as physical activity, sunlight exposure, diet, medication, alcohol consumption, and smoking status on bone metabolism was difficult to evaluate [26]. Finally, bone biopsies were not performed for accurate osteoporosis diagnosis based on low trabecular bone volume and for evaluation of the histological type of renal osteodystrophy.

## 5. Conclusions

Leptin is a significant factor associated with lumbar BMD. Further clinical studies are necessary for an accurate evaluation of the underlying mechanisms.

## Figures and Tables

**Table 1:** Baseline characteristics by categories of bone density in our study.

Characteristics	All patients (*n* = 98)	Normal (*n* = 60)	Osteopenia (*n* = 25)	Osteoporosis (*n* = 13)	*P* value
Age (years)	66.28 ± 9.40	64.15 ± 9.27	69.84 ± 9.33	69.23 ± 7.74	0.017^*∗*^
Hemodialysis duration (months)	56.52 (24.18–110.85)	48.24 (21.63–103.62)	69.84 (26.94–129.48)	80.40 (28.56–123.42)	0.247
Height (cm)	159.27 ± 8.16	161.88 ± 7.87	156.20 ± 7.14	153.08 ± 6.10	<0.001^*∗*^
Prehemodialysis body weight (kg)	62.51 ± 14.71	67.98 ± 14.34	56.62 ± 11.08	48.61 ± 7.83	<0.001^*∗*^
Post-hemodialysis body weight (kg)	60.47 ± 14.34	65.78 ± 13.88	54.82 ± 11.10	46.83 ± 7.73	<0.001^*∗*^
Waist circumference (cm)	90.98 ± 11.92	94.87 ± 11.48	88.02 ± 9.09	78.77 ± 8.99	<0.001^*∗*^
Body mass index (kg/m^2^)	23.70 ± 4.70	25.04 ± 4.68	22.42 ± 4.10	19.97 ± 3.14	<0.001^*∗*^
Lumbar bone mineral density (g/cm^2^)	0.93 ± 0.19	1.05 ± 0.13	0.78 ± 0.07	0.65 ± 0.05	<0.001^*∗*^
Lumbar T-score	-0.71 ± 1.58	0.30 ± 1.03	-1.90 ± 0.55	-3.08 ± 0.49	<0.001^*∗*^
Lumbar Z-score	0.52 ± 1.05	1.08 ± 0.77	-0.07 ± 0.60	-0.92 ± 0.80	<0.001^*∗*^
Systolic blood pressure (mmHg)	141.59 ± 25.55	144.83 ± 24.10	140.48 ± 28.61	128.77 ± 23.51	0.117
Diastolic blood pressure (mmHg)	75.20 ± 15.64	76.45 ± 15.06	74.24 ± 12.21	71.31 ± 23.22	0.532
Total cholesterol (mg/dL)	142.73 ± 33.68	138.77 ± 34.08	147.48 ± 32.48	151.92 ± 33.60	0.320
Triglyceride (mg/dL)	114.50 (86.25–182.50)	132.50 (99.25–199.50)	95.00 (84.00–132.00)	101.00 (59.00–126.00)	0.015^*∗*^
Glucose (mg/dL)	133.00 (111.00–175.50)	141.00 (110.25–192.75)	128.00 (112.50–156.50)	132.00 (108.50–160.50)	0.410
Albumin (mg/dL)	4.10 ± 0.36	4.16 ± 0.36	4.07 ± 0.36	3.88 ± 0.31	0.044^*∗*^
Globulin (mg/dL)	3.13 ± 0.55	3.12 ± 0.46	3.21 ± 0.77	3.07 ± 0.49	0.708
Blood urea nitrogen (mg/dL)	59.58 ± 14.19	59.67 ± 13.81	57.92 ± 14.90	62.38 ± 15.21	0.658
Creatinine (mg/dL)	9.20 ± 1.99	9.51 ± 1.96	8.76 ± 1.96	8.58 ± 2.03	0.139
Total calcium (mg/dL)	9.00 ± 0.67	8.97 ± 0.65	9.21 ± 0.71	8.78 ± 0.61	0.129
Phosphorus (mg/dL)	4.68 ± 1.27	4.80 ± 1.15	4.45 ± 1.42	4.60 ± 1.53	0.504
Intact parathyroid hormone (pg/mL)	185.00 (66.60–372.93)	155.55 (57.53–273.15)	306.70 (71.85–466.90)	291.40 (73.15–553.65)	0.067
Leptin (ng/mL)	11.80 (4.40–53.10)	23.78 (8.41–72.06)	10.92 (3.29–33.70)	1.39 (0.89–2.44)	0.017^*∗*^
Urea reduction rate	0.74 ± 0.04	0.72 ± 0.04	0.75 ± 0.04	0.76 ± 0.04	0.004^*∗*^
Kt/V (gotch)	1.34 ± 0.16	1.30 ± 0.15	1.40 ± 0.17	1.42 ± 0.15	0.004^*∗*^
Female (%)	50 (51.0)	22 (36.7)	16 (64.0)	12 (92.3)	<0.001^*∗*^

Values for continuous variables given as means ± standard deviation and test by the one-way analysis of variance; variables not normally distributed are given as medians and interquartile range and test by the Kruskal–Wallis analysis; values are presented as number (%), and analysis was performed using the chi-square test. ^*∗*^*P* < 0.05 was considered statistically significant. Kt/V, fractional clearance index for urea.

**Table 2:** Clinical characteristics with gender, comorbidity, drugs used, and lumbar bone mineral density levels among hemodialysis patients.

Characteristic		Number (%)	Lumbar BMD (g/cm^2^)	*P* value
Gender	Male	48 (49.0)	1.03 ± 0.16	<0.001^*∗*^
	Female	50 (51.0)	0.84 ± 0.17	

Diabetes	No	51 (52.0)	0.89 ± 0.20	0.052
	Yes	47 (48.0)	0.97 ± 0.18	

Hypertension	No	49 (50.0)	0.91 ± 0.19	0.210
	Yes	49 (50.0)	0.96 ± 0.19	

Hyperlipidemia	No	73 (74.5)	0.92 ± 0.19	0.306
	Yes	25 (25.5)	0.96 ± 0.20	

ACE inhibitor or ARB use	No	77 (78.6)	0.94 ± 0.19	0.579
	Yes	21 (21.4)	0.91 ± 0.20	
*β*-Blocker use	No	66 (67.3)	0.94 ± 0.21	0.747
	Yes	32 (32.7)	0.92 ± 0.16	

CCB use	No	64 (65.3)	0.94 ± 0.20	0.657
	Yes	34 (34.7)	0.92 ± 0.17	

Statin use	No	82 (83.7)	0.92 ± 0.19	0.368
	Yes	16 (16.3)	0.97 ± 0.20	

Fibrate use	No	89 (90.8)	0.93 ± 0.19	0.696
	Yes	9 (9.2)	0.95 ± 0.22	

ACE, angiotensin-converting enzyme; ARB, angiotensin receptor blocker; CCB, calcium channel blocker. ^*∗*^*P* < 0.05 was considered statistically significant. Data are expressed as means ± standard deviation and test by Student's *t*-test.

**Table 3:** Correlation between lumbar bone mineral density levels and clinical variables among hemodialysis patients.

Variables	Lumbar bone mineral density (g/cm^2^)				
	Simple linear regression		Multivariable linear regression		
	*R*	*P* value	Beta	Adjusted *R*^2^ change	*P* value
Female	−0.490	<0.001^*∗*^	−0.256	0.048	0.001^*∗*^
Age (years)	−0.321	0.001^*∗*^	−0.181	0.044	0.012^*∗*^
Log-HD duration (months)	−0.089	0.382	—	—	—
Height (cm)	0.531	<0.001^*∗*^	—	—	—
Pre-HD body weight (kg)	0.570	<0.001^*∗*^	0.219	0.325	0.008^*∗*^
Waist circumference (cm)	0.429	<0.001^*∗*^	—	—	—
Body mass index (kg/m^2^)	0.418	<0.001^*∗*^	—	—	—
Systolic blood pressure (mmHg)	0.105	0.305	—	—	—
Diastolic blood pressure (mmHg)	0.011	0.913	—	—	—
Total cholesterol (mg/dL)	−0.076	0.456	—	—	—
Log-triglyceride (mg/dL)	0.220	0.029^*∗*^	—	—	—
Log-glucose (mg/dL)	0.084	0.409	—	—	—
Albumin (mg/dL)	0.239	0.018^*∗*^	0.148	0.017	0.044^*∗*^
Globulin (mg/dL)	−0.029	0.780	—	—	—
Blood urea nitrogen (mg/dL)	0.037	0.715	—	—	—
Creatinine (mg/dL)	0.220	0.029^*∗*^	—	—	—
Total calcium (mg/dL)	0.005	0.957	—	—	—
Phosphorus (mg/dL)	0.123	0.229	—	—	—
Log-iPTH (pg/mL)	−0.127	0.214	—	—	—
Log-leptin (ng/mL)	0.568	<0.001^*∗*^	0.483	0.184	<0.001^*∗*^
Urea reduction rate	−0.382	<0.001^*∗*^	—	—	—
Kt/V (gotch)	-0.380	<0.001^*∗*^	—	—	—

Data of triglyceride, glucose, iPTH, and leptin levels showed skewed distribution and therefore were log-transformed before the analysis. Analysis of data was performed using the simple linear regression analysis or multivariable stepwise linear regression analysis (adapted factors were gender, age, height, pre-HD body weight, waist circumference, body mass index, log-triglyceride, albumin, creatinine, log-leptin, urea reduction rate, and Kt/V). HD, hemodialysis; iPTH, intact parathyroid hormone; Kt/V, fractional clearance index for urea. ^*∗*^*P* < 0.05 was considered statistically significant.

## Data Availability

The datasets used and analysed during the current study are available from the corresponding author upon reasonable request.
